# Miscellaneous Practice

**Published:** 1870-07

**Authors:** F. B. Norcom


ARTICLE IV.—*Miscellaneous Practice.* By F. B. NORCOM, A.M., M. D.

(Reported by CHARLES CALDWELL, M. D.)

TRAUMATIC PERITONITIS.

CASE 1st. John R., æt. 17, sanguine temperament, and very healthy always, was wounded, May 26th, 1868, by the accidental discharge of a pistol, the ball entering the abdomen three inches to the right of the median line, and two below the plane of the umbilicus. Passing directly backwards it lodged beneath the integument, just three inches to right of spinal column. The accident happening near the Cook County Hospital, Dr. Root was summoned, who took the young man home, and attended him,—dressing the wound, giving full dose morphine, and doing all in his power to relieve his painful condition.

Dr. Norcom, the family physician, arrived at 3 o'clock P. M. and took charge of the case. The shock following the injury was almost a collapse. There was a discharge from the wound of blood and some dark looking fluid, which both Drs. Root and Norcom thought came from the ascending colon; and as he had considerable vomiting, the proof became more positive that the bowel had been injured. Dr. N., after a careful analysis of the case, concluded that his best chance for recovery lay in the full exhibition of opium, carried if necessary to narcotism; and under his directions ½ gr. morphine was given, the pulse being 120, respiration 40, with acute pain in bowels, and ¼ gr. every hour until its influence could be seen, which about 10 P. M. became apparent, as both pulse and respiration began to fall gradually.

Throughout the night he rested tranquilly, his urine being drawn off 10 P. M. and 8 A. M., 27th, when the respiration had fallen to 17 and pulse 110, and throughout the day the opiate was given every three hours, holding the respiration between 16 and 20, the pulse keeping an average of 110.

About 10 P. M., being somewhat nauseated, he vomited, or rather regurgitated four or five ounces of a dark green bilious looking fluid, for which small pieces of ice were given, and as about this time symptoms of exhaustion were being rapidly developed, brandy was ordered every three hours throughout the night, and the hot fomentations of hops assiduously applied, to relieve tenderness and tympanitic distention.

The decubitus was on right side, with abdomen partially resting on the bed, in such a position as to approximate the wounds of abdominal wall and injured bowel, to prevent the escape of fluids in the cavity, and to facilitate soon as possible adhesion, and this position was maintained until inflammation had in a measure subsided. The first 24 hours the abdominal muscles were very rigid, but as distention increased they became more relaxed. On 27th and 28th the abdomen was very much distended, and tender all over from pressure; on the 29th, however, it began to diminish, when the existence of effusion became manifest; and on the morning of the 30th the pain was limited to a space of 3 or 4 inches diameter surrounding entrance of ball, where by this time adhesions were rapidly forming. Early on 31st had very acute pain in track of colon, due probably to distention from flatus, or the passage of hard fæcal matter, which was speedily allayed by warm opiate and aconite fomentations, though during the following night the tympanitis was greater than ever before.

To this date the urine had to be drawn every 12 hours, when he began to regain power, and passed it himself. The opiate treatment had been steadily pursued, with beef-tea and such quantities of brandy as the necessities of the case demanded; but, at end of this day—31st—a sudden change took place, the pulse running up to 140, very feeble and thready, while the respiration fell to 11 per minute and very irregular,—breathing three times in six seconds, with intermission of nine, and as he appeared to be sinking rapidly, brandy was given *ad libitum*, for his extremities were cold and shriveled, and his face bloodless and pinched.

The relatives and friends, supposing him dying, came to utter their farewells, though he lay quiet and lethargic, nor could hardly be aroused by questions put to him; but the stimulant treatment was pushed, and after a few hours the color began to return to his face, the extremities to grow warm, and his rallying became as sudden as his sinking had been.

Having gone to dinner, being absent about one hour and a half, was surprised upon my return to see the sudden change for the better, the pulse falling rapidly to 126, and respiration rising to 16. The mother, during my absence, being very anxious, was imprudent enough to tell him he could never recover, but his courage and hope were proof against such predictions, and he expressed a determination to live through this ordeal, and he did so.

The disease had now reached its acme in the morning, when he came so near slipping through our fingers, and we had now nothing to fear, unless a relapse from imprudence, or the supervention of some such unhappy accident as diarrhœa, vomiting, hiccough, etc., etc. Treatment now gr. $\frac{1}{6}$ morphine every three hours; chicken broth or beef tea; corn starch; milk, etc. Pulse fell quite fast, being, 10 P. M., 106, respiration 18, regular, and sleep unbroken and refreshing during the night. At 7 A. M. pulse 100, respiration 18, and says he feels so well. Saw him again at noon, and was doing finely, but was called in haste at 4 P. M. to find him in great pain, with pulse 100, and respiration 28. This condition seemed to come from an effort on his part to arise from the bed and pass water, during the temporary absence of his mother, and he now feels as if there was to be a change. Lies on his back, with knees drawn up; severe pain in track of colon, and is restless and uneasy. Gave gr. $\frac{1}{2}$ morphine, and at 5.30 P. M. pulse 100, respiration 22; at 6.30 repeated the dose, and at 8 P. M. was asleep, pulse 96, respiration 20. About 11, vomited slightly, and about 12 o'clock, 1st June, pulse 96, respiration 20, perspiring freely, and his breathing slowly falling to 16 by 2.30 A. M., with some occasional vomiting, though accompanied by no pain. From this time he improved slowly, with little vomiting, while at 9 P. M. pulse stood 84, respiration 18, and he now passed for the first time a small purely fœcal stool.

A few weeks later, the ball was extracted by simple incision, and the patient is in the enjoyment of full health.

The record of this case has been little altered, except some slight pruning and condensation, for which I know my friend, the Doctor, will pardon me. The patient undoubtedly owes his life to the skillful and judicious management of Dr. Caldwell; and I can say that, throughout the whole sickness, my own services as consulting physician were of little account, and that everything necessary to meet the emergencies was promptly furnished by this accomplished physician.

During the late war, very many gun-shot wounds of the abdomen were presented for treatment, many including wounds of the large and small intestines; and though, of course, the mortality was great, yet a number of lives were saved—where indeed the chances seemed desperate—by the free use of opium, sustaining with liquid nourishment, and the judicious exhibition of alcoholic liquors. In the same way, penetrating wounds of the chest, especially when the lungs were involved, and attended by severe hæmorrhage, were treated by opium, or its alkaloid, and with beneficial results. For those two large classes of inflammatory affections—serous membranes, and mucous and cutaneous surfaces, the latter two of an erysipelatous character — there exists no remedy that for one moment can compare with it; and in that peculiar condition of the muco-serous lining of the smaller bronchial tubes and air vesicles—bronchitis—known as occurring in such blood diseases as erysipelas, rheumatism, gout, etc., it becomes *par excellence* our sheet anchor; especially, too, if combined with quinine, colchicum, and the ordinary auxiliaries of a sustaining treatment. Witness those attacks of iritis occurring in the later stages of continued fevers, and see how rapidly the effused lymph will disappear under the tonic influence of quinine and small doses of opium, when mercury would be useless to save sight, even if it did not destroy the patient. Again, in those pericardial and endocardial complications following acute rheumatism, especially in younger life, and generally showing themselves the first week —not so very frequent, I grant—observe how certainly they will be controlled by large doses of opium and quinine (*vide* Todd) assisted of course by repeated counter-irritation, and a nourishing dietary. And again, when gout may attack the stomach, giving rise to such acute distress from gaseous accumulation; or the heart, with its frightful intermissions; and ever producing dangerous meningeal irritation, can any drug be found like opium to meet the expectations of the practitioner?

You will not think, Mr. Editor, that there is too much enthusiasm displayed in thus speaking on some of our favorite themes; remember, I do not ride hobbies, as I consider such an infatuation fair evidence of a badly balanced mind; but I am a firm believer that, if this "God-given" drug were taken from us, more than half of our usefulness to the human race would be lost. Our most potent remedies are found in such drugs as act directly on the nervous system,—such as opium, quinine, aconite, digitalis, *et hoc omne genus*,—and hence it is undoubted that our best advances must be in the direction of neuro-pathology. We know that the vaso-motor system of nerves controls the supply of blood, and thus directly affects nutrition; and hence in all inflammatory diseases, where there is dilatation of the blood vessels, whether considered as the primary stage, or simply as a necessary accompaniment to increased cell growth (Virchow,) we necessarily resort to those remedies which, acting upon the sympathetic system, create contraction of those vessels, (Wilkes). Now the remedies above mentioned act in this manner, and few physicians there are who have not observed the remarkable effects of quinine (not anti-periodic) in certain stages of fever; of the action of that blessed anti-inflammatory drug, opium, in the acute diseases of the chest and abdomen; of the manner in which aconite and digitalis control the circulation, thus cutting short in a few hours many diseases, which, if allowed to go on uncontrolled, would, from their severity, cause long periods of confinement, if they did not eventually destroy life.

ALPHOS CIRCINATUS. (Syn: *Psoriasis Vulgaris.*)

CASE 2nd. Mr. H——; æt. 30 years; sanguine temperament, with strongly marked strumous tendencies; unmarried; and lawyer by profession. Habits always regular, never addicted to use of liquor or tobacco. Has been visited by no diseases, not even those incidental to childhood, until the occurrence of his present ailment, which first showed itself in his seventh year. It began to appear at this age just above the ankles, on the fibular surface of both lower extremities in the fall of 1847, and disappeared the following spring and summer, when the functions of the skin became more active.

From that date to June 24th, 1868, when he applied to me, the eruption had been gradually spreading, creeping slowly upwards, involving the abdomen and trunk, the upper extremities, and lastly scalp and face. Up to 1859, it had generally left him through the warm months, the disappearance, however, being each succeeding year less marked, but from this date it took firm hold, and exhibited itself persistently, despite every method of treatment pursued. He states that he was prescribed for by many physicians, sometimes with alleviation, but at others the eruption would return with extreme aggravation.

His digestive system, soon after the first appearance, began to show some derangement, the food seemed to oppress him, giving not only uneasiness, but at times intense pain, paroxysmal and so acute as to double him up like attacks of colic, and then his nutrition became impaired, he began to lose flesh, and his mind so perturbed, that, like most dyspeptics, he grew into absolute hypochondriasis.

His antecedent history is the same as in most of such cases, beginning by a few tubercles capped by the ordinary scale; clusters beginning to develop, fusing into circular masses, then these aggregating into irregular patches upon a slightly elevated dull red base, well marked about the knees and elbows, and attended by the usual itching and desquamation. When he visited me for advice, June 24th, 1868, he was pretty well flecked *à capitè ad calcem*, the eruption perfectly characteristic of the guttated variety on the limbs and body, but so aggregated in patches about the joints of elbow and knee, as to have lost its definite circular outline in a great measure, though not exactly diffuse, with raised borders and depressed centers, and well covered with those imbricated scales of morbid epithelium. The scalp was suffering, the hair being filled with the cast off dèbris, and the forehead and cheeks variegated with many small circular and oval spots of the pityriasic variety. The pruritus was not intense, nor was there at this period any aggravation from such intercurrent visitors as erythema or eczema. Though his general health appeared fair, yet his face and condition indicated nutritive and nervous debility; tongue furred, his digestion pointed to defective gastric secretion; bowels somewhat costive; appetite capricious; and the concomitant mental disturbance sufficient to make him unhappy; to retire him, on account of his facial disfigurement, from society; and, not being in love with Chicago associations, to make him hate his species; and not to be wondered at in any man, no matter how healthy, who is engaged in business in this God-forsaken place.

Recognizing the case in all its bearings, I began treatment by regulating his bowels; assisting the digestion by my favorite combination of Gentian, Nux Vomica, Bismuth, and a small amount of

Morphia; and at meals the arsenio-ferruginous mixture of Wilson. His hair was cut close, the shampoo bath several times a week, with Juniper tar soap; the scalp well rubbed with a white precipitate ointment diluted with glycerine, as also those portions of the eruption where dryness formed a prominent feature, and his dietary limited to nutritious and easily digested substances. Under this judicious treatment, *me judice*, he began to improve slowly but surely, the eruption to clear up from center to periphery of each mass; the morbid epithelial secretion to cease; the pruritus to become less; the digestion to strengthen, with increasing desire for food; and his mental distress so much relieved, that he blessed the day he had applied to me, and I think he was right. About the following January, having passed through a cold snap, and having failed to pursue the treatment as rigorously as advised,—though at this time his skin was well cleaned, as also his face, except some few melasmic stains,—he again fell back, and the disease began to gain ground, and his digestion to again sympathize. Knowing that one's system may become habituated to a certain course of remedies, and that their therapeutical action may become more or less inert, I changed to the acid solution of arsenic (*de Valangin*), reinstated the baths, and ordered a very generous dietary. These measures were faithfully adhered to until the next Autumn, with almost complete disappearance of the affection; a thorough restitution of his gastric functions, and his skin became as pure and healthy as a new born baby's,—barring a few small patches about the joints. And yet, with all this improvement, secured by such protracted treatment, we can hardly hope for a permanent cure, inasmuch as his hereditary proclivities are too direct, and are constantly being lighted up and perpetuated by a too well marked strumous diathesis.

It is an accepted fact, and established by clinical observation, that arsenic is the remedy—almost a specific—in the treatment of the Squamæ. It is a neuro-tonic acting upon the peripheral plexus of nerves, a suspender of waste, and a vital assistant to blood-making. As alphous affections arise from a diathesis, it is to be inferred that, in its administration we desire an alterative effect, and hence give small doses for a long period; at the same time knowing that they are accompanied—even though the patient may be apparently healthy—by great defects of nutrition and sanguification, we combine iron and such nervines as may enhance its own inherent virtues.

The case we have presented exhibits several points of interest; the first, the occurrence of the disease at the early age of seven years, when, as a rule, it is most apt to attack after puberty, or even in middle life, when growth has ceased, and waste often runs ahead of repair; again, its transmission from the paternal grand-father, neither his father, nor any of his brothers or sisters showing the least symptom; and lastly, after existing twenty-one years, how rapidly it improved, and was held in check by treatment. The differential diagnosis is not difficult, as only the tubercular syphiloderma can be mistaken for it. In the latter, the previous history will inform us of certain groups of specific symptoms; the eruption lacks symmetrical diffusion; it is not found around the articulations; and though its duration may be chronic, yet alphos exceeds all known cutaneous affections for chronicity. It is recommended by Wilson—one of the best authorities—to give arsenic in small doses to secure the alterative and tonic effect, but never to push the drug so as to produce constitutional irritation, and to keep up the remedy for months, or years should the necessities of the case demand. He rarely gives more than four or five minims of Fowler's Solution three times a day, or double that number of Valangin's Solution, and if he notices any symptoms of saturation,—or cumulative action (?)—he suspends at once. Arsenic has been tested by myself in a great number of such affections, but I must confess to having never obtained in cases of long standing any remarkable curative effects, until I had induced systemic saturation; the zonal congestive bands of conjunctivæ; perhaps muscular prostration, with nausea and colic; and in certain instances, sub-cutaneous effusion of lids, face, hands, etc. The remedy thus showing its irritative action, I merely lower the dose, say one-third, and soon as possible raise it again to as high a point as the system will bear, of course cautiously and gradually, watching my patient with closest observation. This is my own experience, with most of the arsenical preparations, and it can be safely said, that when the smaller doses—five to six minims—of the Liquor Potassæ Arsenitis produce no effect, given for weeks and months, that by increasing *one* minim every *two* or *three* days, until *ten* or *fifteen* are taken, a most decided improvement will begin to appear; and in no instance have I witnessed any poisonous or deleterious action, nor been obliged to suspend—even though the dose had to be lowered—its administration.

### ACUTE DYSENTERY.

CASE 3rd. Mr. F——; æt. 34; married; nervo-bilious temperament; mason by occupation; and resident of Chicago seven years. Never suffered from any serious disease before. Habits are regular, and is very temperate, both as to the use of liquors or tobacco. Was taken ill Oct. 27th, 1869, and without assignable cause. Came home in the afternoon, feeling very chilly and weak; without appetite; some degree of feverishness; pains in the limbs, back, and abdomen; and throughout the night had several loose diarrhœal discharges. Next day, the 28th, being worse, the discharges becoming more frequent, accompanied by increased abdominal pain and tenderness, called in a physician. A purgative was immediately administered, to be followed by Dover's powder; with proper fomentations to belly; absolute rest in bed; demulcent drinks; and unirritating diet. Despite this judicious treatment, the discharges continued, streaked with blood, and now entirely of a mucous character; while a most distressing nausea and tenesmus came on; great disgust for anything by the mouth; increased tenderness of abdomen by palpation; marked prostration and despondency of mind; and a decided icterode shade of the surface.

On the 2nd Nov., having dismissed his attending physician—a very common occurrence among that class of Irish—he was persuaded by some friend to send for me, and I found him with a pulse 116, very weak and compressible; respiration 28; skin jaundiced and clammy—a condition so happily expressed by the French *poissonneuse;* tongue heavily coated with teeth indentations; abdominal tenderness and distension; much nausea; discharges frequent, scanty, with blood and pus; and decided muscular prostration. Recognizing that his former attendant had done all in his power to control these lesions, and that his treatment had been conducted properly and scientifically; I was obliged either to follow in his footsteps — *secundem artem,* — or to adopt a mode of management used in the extreme Southern States, where dysentery, in the warm months, becomes so prevalent, and either as an endemic or epidemic disorder, at times fearfully fatal.

So, as a *dernier réssort*, he was ordered the infusion of the root of Ipecacuanha, as follows: 2 drachms of the root broken in small fragments, and infused in one pint boiling water, allowed to boil for a few moments, then cooled, and the supernatant fluid poured off, of which one-quarter (4 oz.) was given every fifteen minutes, until the whole was taken; at the same time warm fomentations to the abdomen continued, and absolute rest in the horizontal posture. Of course the most violent emesis ensued, continuing off and on for several hours, assisted by warm drinks and teas, and as soon as this had ceased, iced milk with lime-water was allowed, and at night a full dose of Battley's Sedative. He rested very fairly this night; only two slight discharges, and next morning his condition was improved, though there was still pain and tenderness, with a slight percentage of blood in the last stool, passed just before my morning visit. Upon the same pieces of the root twelve ounces of boiling water were poured, and, after cooling, one-fourth given as in the first instance, and though creating some little nausea and vomiting, it began acting—as it always does—on the bowels, bringing away profuse bilious discharges.

After a few hours the febrile excitement (asthenic) had lessened; the pulse down to 90, softer and fuller; the pain and tenderness almost gone; tongue cleaner; and a ravenous desire for food—this latter a very important sign, and almost pathognomonic of speedy recovery.

He was now permitted cool farinaceous diet; iced milk; warm applications persisted in; and this night 12 grs. Dover's powder, there having been but one small passage since 3 o'clock, P. M., unattended by pain or straining, and no admixture of blood. After this evening no other medication was used; his diet simply regulated; rest still in bed enjoined; and a stimulating camphorated liniment applied to belly, covered by single layer of flannel, to guard against changes of temperature.

On the 6th he was allowed some meat broths, and a moderate amount of claret wine, and simply cautioned against excesses to prevent relapse.

This case is presented simply to call attention to another manner of administering Ipecacuanha in dysenteric diseases, and which has been in vogue many years in the extreme South, where the worst forms and most fatal of this terrible disease exist, and which appears about as near a specific as any treatment ever attained in any class of disorders known, not even excepting the malariæ controlled by the alkaloid of the Jesuit's bark. I am well aware that several modes of administering this drug in the same disease have been brought before the profession, have had their advocates, and been sustained by a long list of remarkable recoveries; yet permit me to remark, that I have tried most of them, and can find nothing for one moment to compare with that above detailed. Of course it is unnecessary, especially in these northern climates, to resort to it in the great majority of cases, most of which may be readily controlled by the usual treatment—opiate, astringent, or otherwise; but in those cases where such remedies fail to respond, and where the patient is succumbing to the violence of the disease, it offers at least one more hold, and if employed fearlessly and vigorously will not disappoint the practitioner. It can be used for children, as well as adults, of course modifying the dose and quantity; and even among sucklings, where there seemed few hopes of recovery, life has been saved.

It is the same treatment as that employed in Martinique, the "Leeward Islands," and tropical latitudes generally, and was introduced into Louisiana by a French physician during the Spanish *régime*, and has become so popular, and so well appreciated that, on the large estates, the planters, taking time by the forelock, have all their cases well in hand before the attending physician can see them. During the occupation of Louisiana, when the provost camps were established in the "coast country," the Federal soldiers would indulge in green corn, unripe fruits and sugar cane, shrimps, fish, etc., washing down such complex messes with commissariat, or fighting whisky, and as was to be expected, few escaped violent attacks of bloody flux. As I had charge of one large camp—part of 31st Mass. regiment—this treatment was invariably inaugurated, and though it rather astonished the strangers to be put through such an apparently violent course of medication, yet, with one exception, and that the result of neglect and carelessness, not one single death occurred, while eight miles below, where many were ill with the same complaint, they were carted out by the score to their last resting place in the bosom of mother earth.

I am sure many a poor fellow, who was "sick unto the death," and who expected to leave his bones in that "far away distant land of the orange and myrtle," often thanks his stars, and as often thinks of the rebel doctor—so they termed me—who sat beside him administering a most awful puking, and trying to console and philosophize with him, while he was expecting every moment to be turned inside out, and throw up his very boots—figuratively speaking; and, at the same time, so great a reputation for success had this treatment obtained, that many came in later days (when they could secure furloughs), even from other parts, to be cured of their sub-acute and chronic varieties, for which it was hardly admissible.

And now, as to the *rationale* of treatment, or the *modus operandi* of this drug, I have not one word to say, leaving the whole to the wisdom and philosophical reasonings of the many readers of the JOURNAL; whether they will not place such effects, in part, to the known properties of ipecacuanha in controlling inordinate and abnormal mucous secretions; or whether due to rapid and forcible elimination through emesis. I only give the facts, gleaned from a large experience—and a happy one too—and at the same time must confess that, throughout, the treatment can in no way be called otherwise than empirical.

